# Azure A Dye Interaction with Double-Stranded DNA Can Be Modulated by the Ionic Strength: Choosing the Binding Mode

**DOI:** 10.1021/acsomega.5c09975

**Published:** 2026-02-13

**Authors:** Arthur G. S. de Rezende, Ethe A. Portilho, Josiane A. D. Batista, Márcio S. Rocha

**Affiliations:** Departamento de Física, 28120 Universidade Federal de Viçosa, Viçosa, Minas Gerais 36570-900, Brazil

## Abstract

We study the interaction of the Azure A dye with double-stranded DNA in phosphate-based buffers under three different ionic strengths, showing that the effective binding mode can be modulated by this characteristic of the surrounding buffer. For relatively high ionic strengths ([Na^+^] ≳ 10 mM), groove binding is the preferable mode, while intercalation dominates for [Na^+^] ≲ 1 mM. Single-molecule force spectroscopy assays were performed to determine these binding modes and also to extract the relevant binding parameters of the interaction in each case, resulting in a robust characterization of the complexes formed under different experimental conditions. Furthermore, gel electrophoresis assays were also performed to confirm the main conclusion about the binding mode changes. The present work advances in the characterization of DNA interactions with complex ligands, bringing new insights into the rational development of new synthetic nucleic acid dyes and drugs.

## Graphical abstract

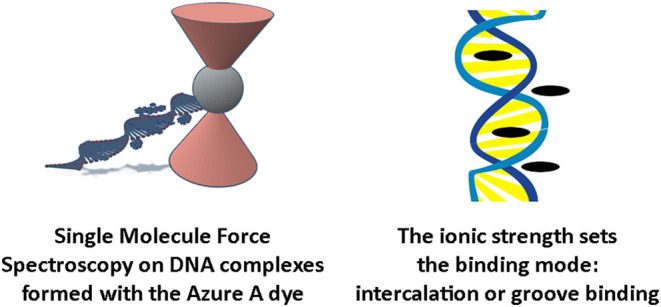

## Introduction

Nucleic acid dyes are in general small molecules used to allow the detection, visualization, and quantification of these biopolymers in various types of experiments such as in fluorescence microscopy, gel electrophoresis, and real-time PCR, to cite a few.^1–8^ DNA dyes, for instance, can bind to the double helix mainly by two modes: intercalation and groove binding, depending on the specific chemical structure of the ligand and on the experimental conditions of the surrounding buffer.^9,10,10–16,16–23^ A very important aspect in studying DNA interactions with small ligands, such as dyes and drugs, is to determine the specific binding modes, the conditions at which these modes occur, and the relevant physicochemical (binding) parameters that characterize the interactions. Such a type of knowledge allows the rational development of more efficient synthetic molecules for a variety of purposes. In the past years, many groups have focused on characterizing DNA interactions with small ligands.^24–34^

In the present work, we characterize the interaction of the Azure A dye (Figure 1) with double-stranded DNA at the single-molecule level for the first time, by performing single-molecule force spectroscopy assays with optical tweezers. Azure A is a synthetic dye that belongs to the phenothiazine class of dyes, widely used in biology and histology. It is structurally related to methylene blue and can be used in various cell staining techniques, particularly nucleic acid staining. Furthermore, Azure A has presented promising results in photodynamic therapies.^35,36^

**Figure 1:**
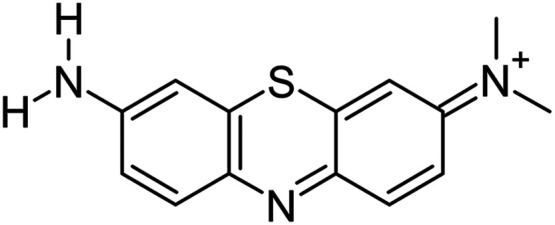
Chemical structure of Azure A dye.

Studies concerning the interaction of Azure A with DNA are very scarce. Paul and Kumar used bulk spectroscopic techniques (UV–visible absorbance, fluorescence quenching, fluorescence polarization anisotropy, circular dichroism) to determine that, under the conditions used, Azure A intercalates DNA in a 50 mM sodium cacodylate buffer, pH 7.2, with an association binding constant on the order of 10^5^ M^–1^.^37^ A similar conclusion was also found by the same authors under the same conditions using microcalorimetric techniques.^38^ Kumar et al. also reported similar results using various bulk techniques such as ultraviolet (UV) absorbance, thermal melting, fluorescence, and circular dichroism for calf thymus DNA in a sodium cacodylate with pH = 7.4, 0.1 M NaCl and 0.1 mM EDTA.^39^ On the other hand, some electrochemical studies report that, besides intercalation, electrostatic interactions between Azure A and the negative phosphate backbone of the DNA double helix are also important,^40,41^ which may lead to another binding mode depending on the experimental conditions.^9^

In fact, our single-molecule studies performed under three different buffers have demonstrated that the binding mode of the Azure A dye to the DNA double helix can be both intercalation and minor groove binding depending on the ionic strength of the buffer. Figure 2 illustrates the difference between these two binding modes on the double helix. Such a type of behavior was previously verified for other complex small ligand that interacts with double-stranded DNA—the drug chloroquine, for which the specific binding mode depends not only on the ionic strength but also on the specific buffer composition, which makes a lot of difference in that case to determine the dominant binding mode.^42,43^ Therefore, the results of the present work allow one to conclude that the Azure A dye is also a complex and intricate DNA ligand despite its relatively simple chemical structure. Furthermore, the present work advances in the characterization of DNA interactions with complex ligands, bringing new insights into the rational development of new synthetic nucleic acid dyes and drugs.

**Figure 2:**
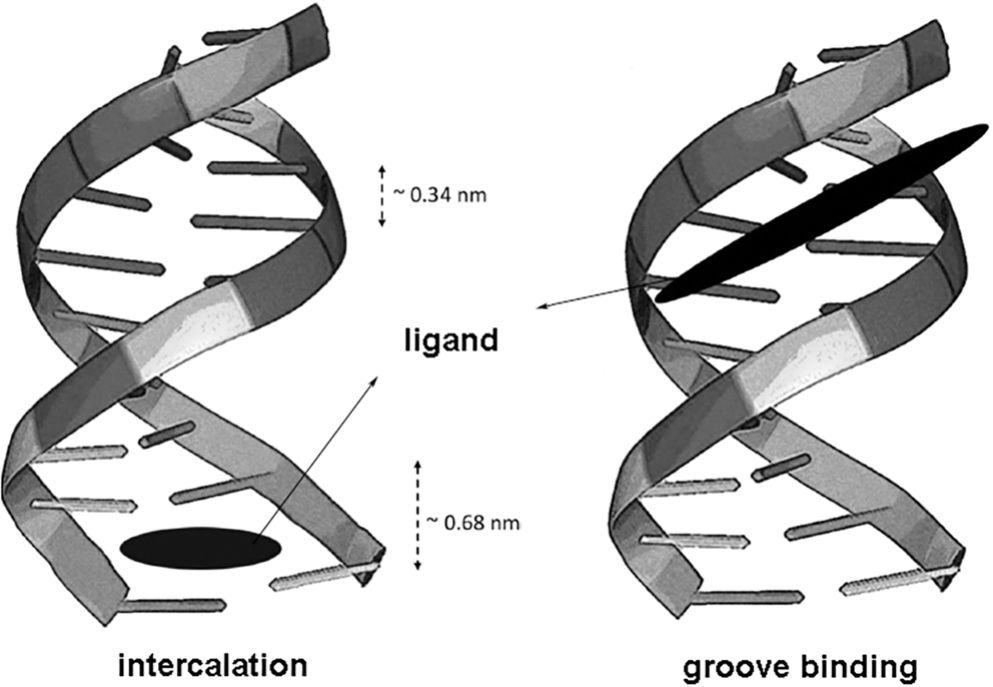
Difference between the two binding modes that usually occur for dye molecules on the DNA double helix. In intercalation, the ligand is inserted between adjacent base pairs, increasing the interspace distance (typically doubling such a distance). The main driving forces associated with this type of binding are π-stacking, hydrophobic interactions, and sometimes hydrogen bonds and electrostatic attraction.^9^ In groove binding, on the other hand, the ligands bind outside the double helix, fitting to the grooves along the double-helix structure. In this case, the main driving forces are electrostatic attraction, hydrogen bonds, van der Waals forces, and sometimes hydrophobic interactions.^9^

## Materials and Methods

### Sample Preparation and Experimental Procedure for Optical Tweezers Assays

Samples prepared for single-molecule stretching experiments consist of mixing previously biotin-labeled λ-DNA (New England Biolabs) molecules with streptavidin-coated polystyrene beads (Bangs Laboratories). Using a standard protocol,^9^ one end of the DNA is attached to a bead and the other end to the surface of a streptavidin-coated glass coverslip, used to construct the sample chamber. The optical tweezers are used to trap the bead, and by moving the microscope stage relatively to the laser, one can stretch the DNA and measure the force–extension curves (FECs) when different concentrations of the Azure A dye are added to the sample chamber. Such procedures were exhaustively used and reported in previous references from our group and proved to work very well for experiments in which one wants to study the changes in the mechanical response of the DNA molecule as a function of the ligand concentration in the sample.

The FECs collected can be fitted to the worm-like chain (WLC) model for the determination of the mechanical parameters of the double helix and its complexes formed with ligands, in our case, the Azure A dye, whose chemical structure is shown in Figure 1. Here, we deliberately use maximum stretching forces on the order of only ∼2 to 3 pN to work in the entropic regime, where the DNA mechanics can be effectively described by only two parameters: the contour and persistence lengths.^9^ Thus, the Marko–Siggia WLC equation^44^ can be used to fit the experimental data and determine these parameters with a high accuracy.^9^ Furthermore, the use of these very low forces also guarantees that no cumulative damage is done to the DNA molecules used during the entire experiment.

The average results reported in the next section for the mechanical parameters as a function of the dye concentration in the sample were obtained by performing repeated stretching experiment assays under the same conditions with the same DNA molecule (5 to 7 independent measurements), and the error bars were calculated as the standard error of the mean. We start this procedure with a bare DNA molecule, perform the stretchings, and then add the smallest dye concentration. After waiting 30 min for ligand equilibration, we repeat the stretchings and then follow this same procedure, gradually increasing the Azure A concentration in the sample, obtaining the graphs representing the changes of the mechanical parameters (contour and persistence lengths) as a function of the dye concentration.

This complete assay was performed under three different ionic strengths using phosphate-based buffers (PBS) whose compositions are detailed in Table 1, all with pH = 7.4. Such a study was made to verify the influence of the ionic strength of the surrounding buffer on the effective binding of Azure A to DNA. Finally, for each different ionic strength used, at least three different samples were analyzed to check the reproducibility of the results.

**Table 1:** Composition of the Different PBS Buffers Used, All with pH = 7.4

[Na^+^]	ionic strength	NaCl	Na_2_HPO_4_	NaH_2_PO_4_
150 mM	154 mM	140 mM	4.375 mM	1.25 mM
10 mM	14 mM	0	4.375 mM	1.25 mM
1 mM	1.4 mM	0	0.4375 mM	0.125 mM

Azure A dye was first prepared as stock solutions of 100 μM in each used buffer. These stocks were stored in boxes sealed with aluminum foil in a fridge and used for a maximum of 15 days, avoiding the photodegradation of the dye. The aliquot solutions at each desired dye concentration were prepared by simple dilution immediately before use during the experiments. They are introduced in the sample chamber containing the DNA molecules and allowed to equilibrate for 30 min before performing the assays.

### Determining the Physicochemical (Binding) Parameters of the Binding Modes

Binding models were previously developed to fit the experimental data of the mechanical parameters of DNA complexes formed with small ligands, allowing one to determine the relevant binding parameters of the effective interactions. A detailed description of these models can be found elsewhere,^9,25,45^ with the complete mathematical details. Here, we will briefly discuss them since they will be used to fit the optical tweezers data in the following section.

The contour length *L* of DNA complexes formed with intercalating ligands presents a very characteristic monotonic increase as a function of the concentration of the bound ligand. The relative increase of this parameter can be modeled with the function Θ = (*L* – *L*_0_)/*L*_0_, where *L*_0_ is the initial contour length corresponding to the bare DNA, i.e., without any ligand. It is straightforward to show that Θ can be expressed for intercalators as^9,25^

1 $$C_{T}=\frac{C_{\mathrm{bp}}}{\gamma }\Theta +\frac{\Theta {\left(\gamma -N\Theta +\Theta \right)}^{N-1}}{K{\left(\gamma -N\Theta \right)}^{N}}$$

where *C*_T_ is the total ligand concentration in the sample, *C*_bp_ is the DNA base-pair concentration, γ is the ratio between the elongation in the contour length promoted by a single bound molecule and the natural base-pair interspace (typically ∼1 for monointercalators), *N* is the binding site size (exclusion number), which express the effective number of base pairs occupied by a bound molecule, and *K* is the equilibrium association binding constant.

This model is based on original the McGhee–von Hippel binding isotherm,^46^ which is the best isotherm to describe the intercalative binding of small ligands on DNA.^9,25^ Eq 1 can be used to fit the experimental data of the contour length as a function of the ligand concentration in the sample, which is the typical type of data that we obtain here with our single-molecule assays.

The persistence length *A* of the DNA complexes formed with small ligands as a function of the ligand concentration in the sample can also be modeled, in this case using a quenched-disorder statistical model. The model is based on calculating the effective persistence length of a polymer partially covered by ligands—an association of “entropic springs”.^9^ The rigorous mathematical demonstration of the model can be found in Siman et al.^45^ For monotonic increases or decreases of the persistence length as a function of the ligand concentration, the model can be written as^9,25^

2 $$\frac{1}{A}=\frac{1-r/r_{\max}}{A_{0}}+\frac{r/r_{\max}}{A_{1}}$$

where *r* is the fraction between the bound ligand concentration *C*_b_ and the DNA base-pair concentration *C*_bp_ (i.e., *r* = *C*_b_/*C*_bp_), *r*_max_ is its saturation value, *A*_0_ is the bare DNA persistence length, and *A*_1_ is the persistence length at bound ligand saturation.

The bound ligand fraction *r* can be connected to an appropriate binding isotherm. For noncooperative interactions, the McGhee–von Hippel isotherm given by eq 1 with Θ = *γr* can be used.^9,25^

### Gel Electrophoresis Assays

Gel electrophoresis competition experiments were also performed to investigate the possible binding modes between Azure A and DNA in different buffers. We used agarose gels with 1.0% mass and a Tris-acetate-EDTA (TAE) 10× solution. The wells in the gel were filled with samples containing DNA and Azure A at chosen concentrations diluted in two of the PBS buffers used in the optical tweezers assays ([Na^+^] = 150 mM and [Na^+^] = 1 mM, the extreme cases). Such samples were previously equilibrated for 30–40 min before the assays. The DNA concentration was maintained fixed, while the Azure A concentration increased for different wells, allowing one to verify the effects of the dye in a wide range of concentrations.

The assays were carried out using the plasmid PBR322 double-stranded DNA (Thermo Fischer). The runs were performed at 78 V for 20 min. Before performing the runs, fluorescent GelRed dye is introduced into the wells. Finally, after the runs, the gels are revealed in a UV transilluminator.

## Results and Discussion

### Single-Molecule Force Spectroscopy with Optical Tweezers

In Figure 3, we show some representative force–extension curves (FECs) for the three ionic strengths used and some Azure A concentrations: (a) [Na^+^] = 150 mM, (b) [Na^+^] = 10 mM, and (c) [Na^+^] = 1 mM, along with the fittings performed with the WLC model (solid lines). Observe that the WLC model accurately fits the experimental data in all situations, allowing one to determine the mechanical parameters (contour and persistence lengths) with high accuracy for each dye concentration used in the sample.

**Figure 3:**
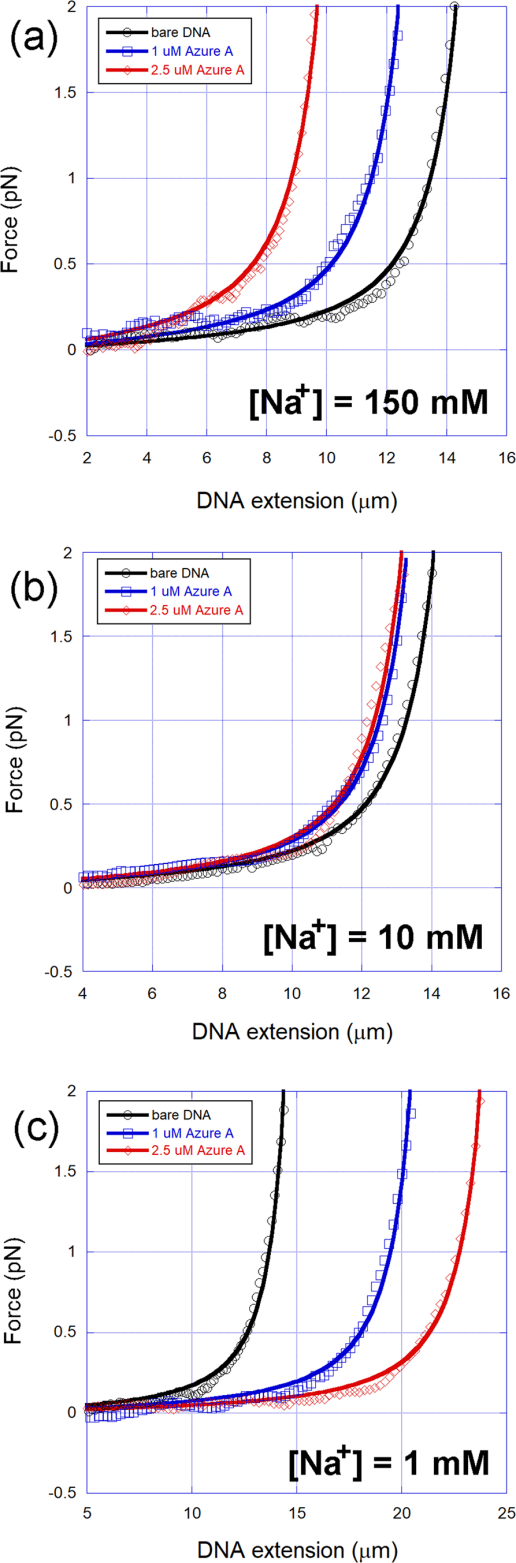
Representative force–extension curves (FECs) for the three ionic strengths used and some Azure A concentrations: (a) [Na^+^] = 150 mM, (b) [Na^+^] = 10 mM, and (c) [Na^+^] = 1 mM, along with the fittings performed with the WLC model (solid lines).^44^

In Figure 4, we show the measured contour length of the complexes formed between Azure A and double-stranded DNA in the three different ionic strengths used: (a) [Na^+^] = 150 mM, (b) [Na^+^] = 10 mM, and (c) [Na^+^] = 1 mM. It is worth noting that the behavior of this mechanical parameter as a function of the dye concentration changes drastically when decreasing the ionic strength of the surrounding buffer. At [Na^+^] = 150 mM, it presents a very prominent decay from the bare λ-DNA value (∼16.5 μm) to only ∼11.5 μm. At [Na^+^] = 10 mM, such decay is much more discrete, and in fact, the contour length remains almost constant within the error bars. Finally, at [Na^+^] = 1 mM, the behavior changes even qualitatively, with the contour length now increasing with the dye concentration.

**Figure 4:**
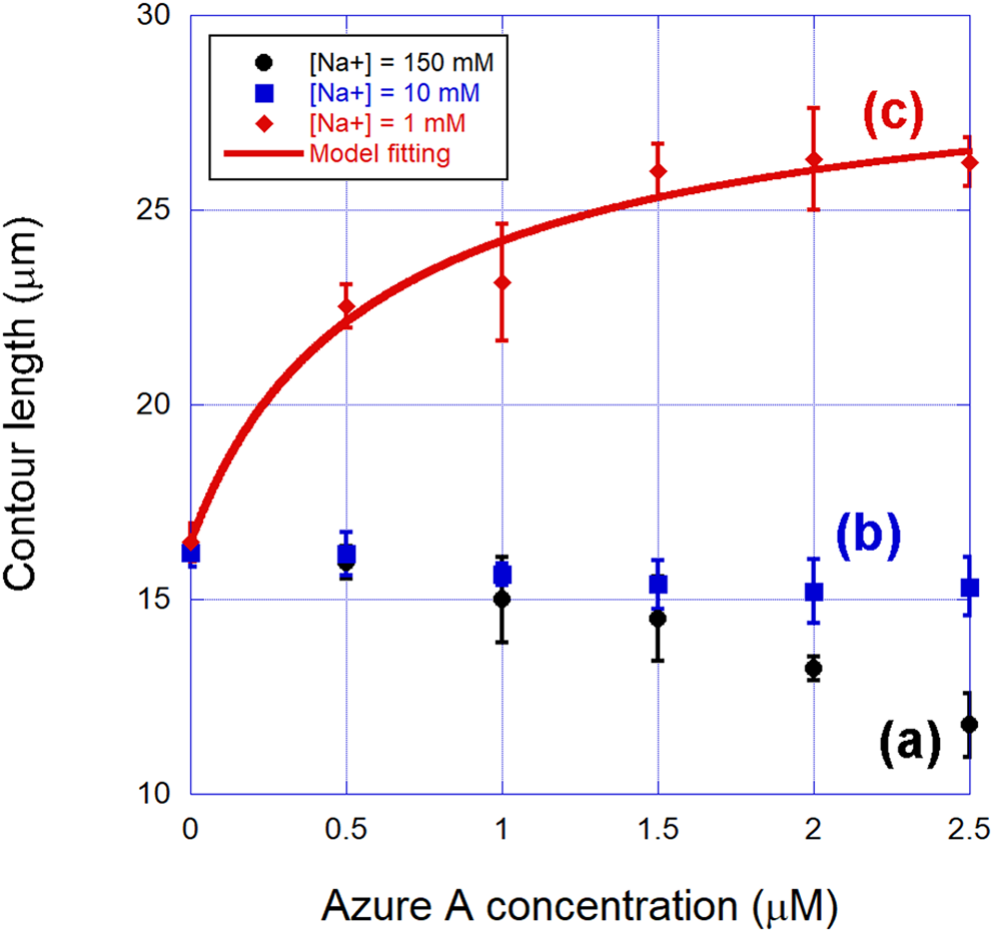
Contour length (apparent) of the complexes formed between Azure A and double-stranded DNA in the three different ionic strengths used: (a) [Na^+^] = 150 mM, (b) [Na^+^] = 10 mM, and (c) [Na^+^] = 1 mM. The behavior of this mechanical parameter as a function of the dye concentration is highly dependent on the ionic strength of the surrounding buffer. The fitting shown in curve (c) was performed with eq 1.

To interpret such pronounced differences, first we need to draw attention to the fact that the measured contour lengths presented here are apparent values of this mechanical parameter since we have limited the maximum stretching forces to ∼3 pN, within the entropic regime. Although such forces are sufficient to almost fully stretch the bare λ-DNA molecule and to determine its nominal contour length with accuracy (see Figure 4 corresponding to zero dye concentration), when one uses a ligand that can promote some DNA compaction, such forces sometimes are not sufficient to fully stretch the complexes formed,^47^ and the WLC fittings return an “apparent” value of the contour length in these cases. One could then ask why we do not increase the maximum stretching forces in order to fully extend the complexes. Although such an experiment is possible, our intention here was exactly to characterize the DNA compaction promoted by Azure A in higher ionic strengths and compare such behavior with the case found for very low ionic strengths, in which there is no compaction, and the contour length indeed increases as the dye molecules bind to the double helix. Thus, for an accurate comparison, one should work within the same force regime in all cases. Furthermore, and more critically, increasing the forces beyond 5 pN can lead to elastic (enthalpic) contributions for the mechanical behavior of the complexes formed between the dye and the DNA molecule, making comparisons more difficult and less accurate.^9^

Therefore, the results shown in Figure 4, curves (a) and (b), explicitly show that, at relatively high ionic strengths, Azure A promotes a compaction of the double-helix structure upon binding. This effect is compatible with a groove binding mode, where the ligand molecules bind outside the double helix at the DNA grooves due to a combination of various intermolecular forces, and was previously reported for other types of similar ligands.^9^ In the case of the Azure A dye, which is a cationic ligand (Figure 1), Coulomb attraction with the negative phosphate backbone of the double helix should play an important role. In addition, the effective interaction should also occur with the contribution of van der Waals forces and/or hydrogen bonds, very common in the minor groove binding of small ligands to DNA.^9^ In any case, the compaction verified should be related to the ligand accommodation along the double helix, which can induce bends toward the binding sites, resulting in an apparent reduction of the contour length when the complexes are stretched using only very small forces. Such bends should also be reflected in an effective decrease of the persistence length of the complexes formed, a result that we really have found here for the two higher ionic strengths and will be presented in Figure 5.

**Figure 5:**
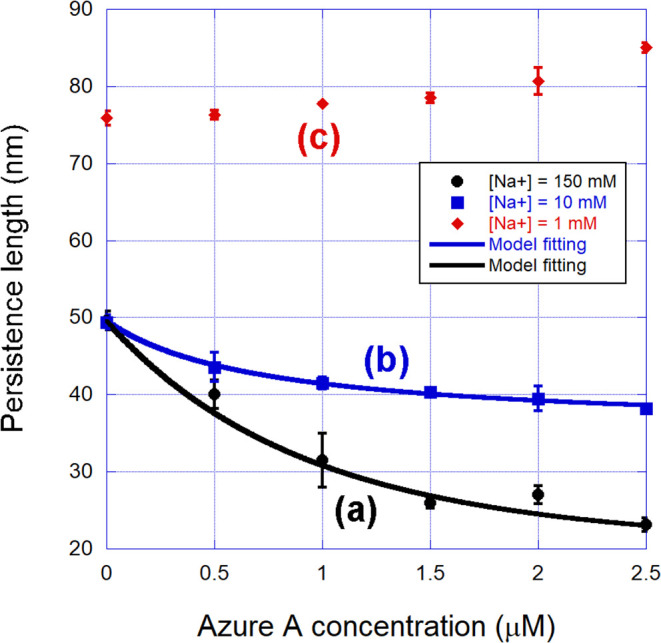
Persistence length of the complexes formed between Azure A and double-stranded DNA in the three different ionic strengths used: (a) [Na^+^] = 150 mM, (b) [Na^+^] = 10 mM, and (c) [Na^+^] = 1 mM. The behavior of this mechanical parameter as a function of the dye concentration is highly dependent on the ionic strength of the surrounding buffer. Note that the persistence length of the bare DNA is around 50 nm for the two higher ionic strengths, but considerably greater (∼76 nm) for the lowest ionic strength used here, in agreement with previous studies.^48^ The fittings shown in curves (a) and (b) were performed with eq 2, using also eq 1 with Θ = *γr*.

On the other hand, the result shown in Figure 4, curve (c), strongly suggests that at very low ionic strengths, there is another dominant binding mode, since the behavior of the contour length was opposite to that verified previously for the higher ionic strengths. In fact, the monotonic increase of the contour length verified in Figure 4(c) is very similar to that typically found for classic DNA intercalators, which bind by inserting their aromatic rings between adjacent base pairs, effectively increasing the DNA contour length.^9,49–52^ In order to advance in such an analysis, we fitted the experimental data shown in Figure 4(c) to the contour length model for intercalators previously discussed. This fitting is shown as a red solid line in the figure. Observe that the agreement between the experiments and the model is very good, allowing one to determine the relevant binding parameters with accuracy, which in this case are the equilibrium association binding constant *K* and the binding site size (exclusion number) *N*. We found here *K* = (1.2 ± 0.2) × 10^6^ M^–1^, *N* = (1.4 ± 0.1), and γ = 1.0 ± 0.1. These parameters are within the range expected for monointercalators (*K* ∼ 10^5^–10^6^ M^–1^, *N* ≳ 1.5, γ ∼ 1.0),^9,53,54^ confirming that this is indeed the binding mode. In particular, the fact that *N* > 1 evidences the well-known neighbor exclusion effect typically exhibited by intercalators, which effectively occupies more than one base pair on their binding sites.^9,46^

In principle, it is possible to model a decrease in the contour length associated with changes in the local double-helix structure.^26–28^ Even eq 1 could be used, supposing a shrink associated with a negative value of γ. However, here we use the persistence length data with the quenched-disorder model presented in the former section, since the interpretations of the physical meaning relative to the results obtained with this approach are very well established.^9^

Therefore, to advance our analysis in Figure 5, we show the corresponding persistence lengths of the same complexes shown in Figure 4, in the same ionic strengths. Observe that for the two higher ionic strengths, curves (a) and (b), the persistence length presents a monotonic decrease as a function of the dye concentration in the sample. For the lowest ionic strength used, on the other hand, such a mechanical parameter exhibits an increase with the dye concentration, which is also typical of intercalators,^20,55–59^ definitively confirming that this is the dominant binding mode at very low ionic strengths.

The persistence length data obtained for the two higher ionic strengths, shown in curves (a) and (b) of Figure 5, on the other hand, can be fitted to the quenched-disorder statistical model presented in the Materials and Methods Section, allowing the determination of the binding parameters in these cases. We have also used the McGhee–von Hippel binding isotherm in such a model, in order to accurately compare the parameters obtained with those determined from the contour length data for the lowest ionic strength. For [Na^+^] = 150 mM, we obtained *K* = (8.5 ± 0.7) × 10^5^ M^–1^ and *N* = (0.48 ± 0.05). For [Na^+^] = 10 mM, *K* = (8.7 ± 0.8) × 10^5^ M^–1^ and *N* = (1.1 ± 0.1). These parameters are within the expected range for groove binders (*K* ∼ 10^6^–10^8^ M^–1^, *N* ≲ 1).^9^ Observe especially that in these cases, we found *N* ≲ 1, different from intercalators. In particular, the result found for *N* at [Na^+^] = 150 mM indicates that two dye molecules are bound per base pair (1/0.48 ∼ 2), suggesting dimerization—a result very common for monocationic dyes at high ionic strengths due to the screening of the electrostatic repulsion between the molecules, which favors π-stacking aggregation.^42,59,60^

In Table 2, we show the results obtained for the binding parameters in all situations, allowing better visualization and direct comparison of such data.

**Table 2:** Binding Parameters Obtained from the Optical Tweezers Data for the Azure A Interaction with Double-Stranded *λ*-DNA at Three Different Ionic Strengths^a^

[Na^+^] (mM)	*K* (×10^5^ M^–1^)	*N*	γ	*A*_0_ (nm)	*A*_1_ (nm)
1	12 ± 2	1.4 ± 0.1	1.0 ± 0.1	-	-
10	8.7 ± 0.8	1.1 ± 0.1	-	49.4 ± 0.5	36 ± 3
150	8.5 ± 0.7	0.48 ± 0.05	-	49.6 ± 0.5	19 ± 2

a The local persistence lengths *A*_0_ and *A*_1_ obtained are also shown for reference.

In summary, the results along with the quantitative analyses performed allowed us to show that Azure A is a complex DNA ligand, and the binding mode can be modulated by changing the ionic strength of the surrounding buffer. For higher ionic strengths ([Na^+^] ≳ 10 mM), minor groove is the preferential binding mode, while intercalation dominates for very low ionic strengths ([Na^+^] ≲ 1 mM).

From the point of view of the binding affinity, in the present case, both modes occur with a similar equilibrium binding constant, on the same order of magnitude (∼10^6^ M^–1^). The main difference is that at higher ionic strengths, the effective binding site size tends to be smaller, with more bound ligands per base pair—a result associated with dye–dye aggregation when the electrostatic repulsion between these molecules is considerably screened by the counterions present in the buffer. Furthermore, while intercalation increases the measured contour length due to ligand accommodation between adjacent base pairs, groove binding in this case decreases this parameter, which can be associated with bends introduced along the double helix on the binding sites.^9^ Other complementary techniques can be used in the future to help distinguish these two different binding modes: absorption and fluorescence spectroscopies, to determine changes in the helix structure; atomic force microscopy, to verify the changes in the contour length via imaging; nanocalorimetry, to study the energetics of the binding modes; among others.

### Gel Electrophoresis Results

Figure 6 shows a typical result obtained from our gel electrophoresis assays. In panel (a), upper line, we show the DNA–Azure A samples in the PBS [Na^+^] = 150 mM buffer, while in the bottom line, we show the samples in the PBS [Na^+^] = 1 mM buffer. The Azure A concentration increases from left to right, as indicated in the figure. Observe that in the bottom line, the decrease of the GelRed fluorescence is more pronounced, indicating a stronger competition between Azure A and GelRed in this situation. Since GelRed is also an intercalating dye,^21^ this result suggests that, in the PBS [Na^+^] = 1 mM, Azure A should present a stronger intercalative behavior, directly competing with GelRed for the binding sites. Such a conclusion agrees with that obtained from the single-molecule experiments: at lower ionic strengths, Azure A prefers to intercalate along the double helix. When the ionic strength is increased, on the other hand, the preferential binding mode changes to groove binding, and the competition with GelRed for binding sites along the double helix weakens, leading to a more pronounced fluorescence (associated with bound GelRed) as can be noted in Figure 6, especially for higher Azure A concentrations.

**Figure 6:**
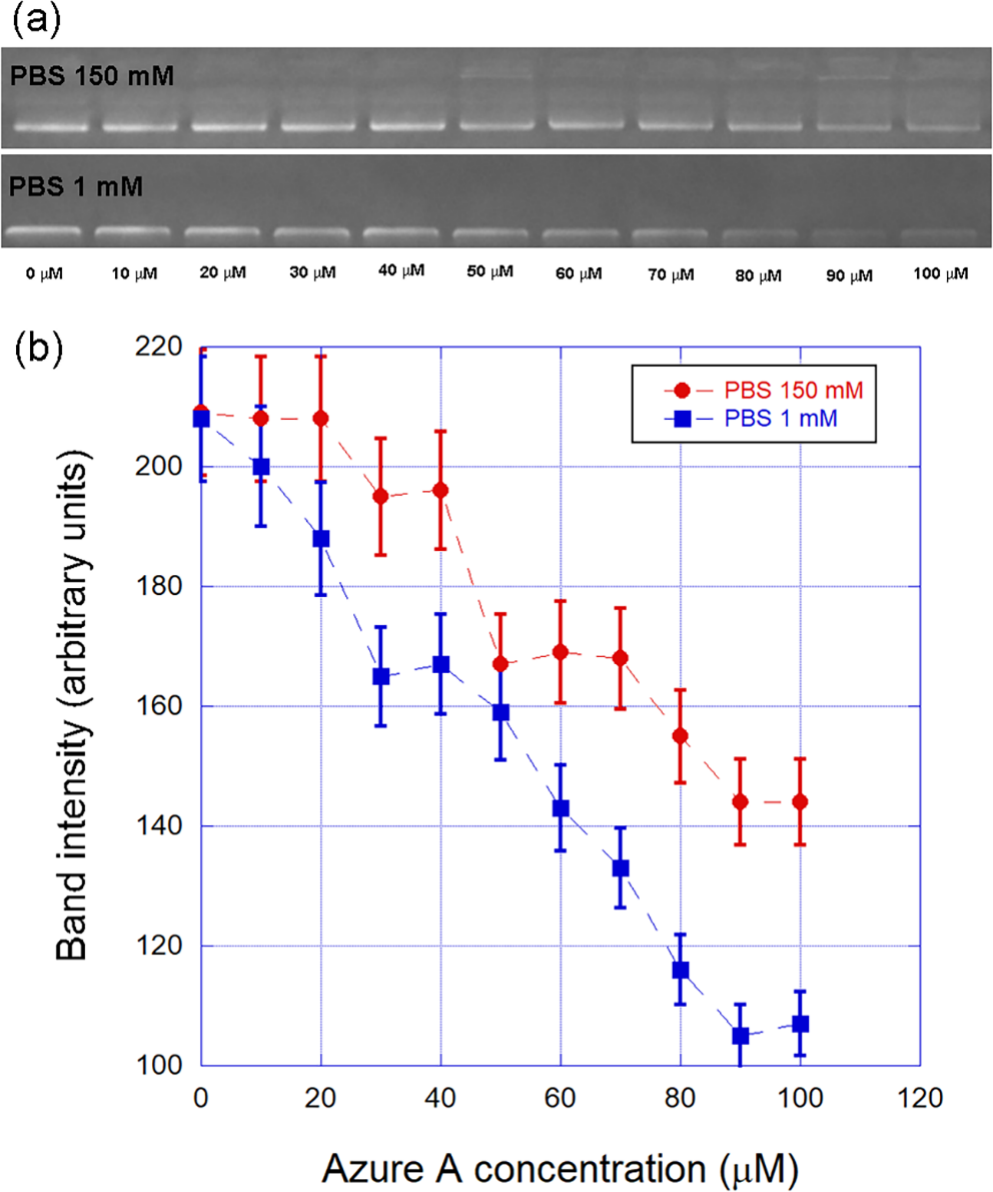
Typical result obtained from gel electrophoresis assays. (a) DNA–Azure A samples at increasing dye concentrations in the PBS [Na^+^] = 150 mM buffer (upper line) and in the PBS [Na^+^] = 1 mM buffer (bottom line). (b) Measured band intensity as a function of the Azure A concentration for the two buffers. These results confirm that Azure A tends to intercalate at lower ionic strengths, competing with GelRed for the intercalation sites along the double helix under this situation.

To better visualize such an effect, in panel (b) of Figure 6, we show the band intensity as a function of Azure A concentration for the two buffers, clearly demonstrating the tendency of the dye to intercalate at lower ionic strengths. The intensities of the bands were measured using the ImageJ software.

## Conclusions

We show that Azure A is a complex DNA ligand that exhibits a subtle competition between two different binding modes with the biopolymer in phosphate-based buffers: while a minor groove dominates for relatively high ionic strengths ([Na^+^] ≳ 10 mM), intercalation is the preferable binding mode in the opposite limit ([Na^+^] ≲ 1 mM). Curiously, the effective equilibrium binding constant increases only ∼40% when decreasing the ionic strength by 2 orders of magnitude, from ∼10^2^ mM to ∼10^0^ mM. The binding site size, on the other hand, increases ∼3 times under the same conditions, which is associated with the change in the dominant binding mode. These results allowed us to show that Azure A is another complex DNA ligand, for which the binding mode can be modulated by changing the ionic strength of the surrounding buffer, an aspect that was not previously reported in the literature at the single-molecule level. The present work thus advances in the characterization of DNA interactions with complex ligands, bringing new insights to the rational development of new synthetic nucleic acid dyes and drugs.
